# Long-term Follow-up of Patients receiving Intraocular Pressure-lowering Medications as Cataract Surgery Candidates: A Case-control Study

**DOI:** 10.5005/jp-journals-10028-1234

**Published:** 2017-10-27

**Authors:** Georgios Bontzos, Michail Agiorgiotakis, Efstathios T Detorakis

**Affiliations:** 1 PhD Student, Department of Ophthalmology, University Hospital of Heraklion, Crete, Greece; 2 PhD Student, Institute of Vision and Optics, University of Crete, Crete, Greece; 3 Associate Professor, Department of Ophthalmology, University Hospital of Heraklion, Crete, Greece

**Keywords:** Aqueous humor, Beta-antagonists, Cataract, Glaucoma, Prostaglandins.

## Abstract

**Aim:**

In this study, we reviewed demographics and biometric characteristics among patients receiving chronic β-blockers and prostaglandins (PGs) for primary open-angle glaucoma. We compared the age at the time of cataract surgery in different patient groups and in a control group which was not under any medication.

**Materials and methods:**

Retrospective chart review of glau-comatous patients who underwent cataract extraction at the Department of Ophthalmology of the University Hospital of Heraklion, Crete, Greece, between January 1998 and December 2016 was done. Age at cataract surgery, axial length (AL), and preoperative and postoperative best-corrected visual acuities (BCVAs) were recorded. A cohort of patients without glaucoma who were operated for cataract extraction was also evaluated.

**Results:**

In all, 320 patients were reviewed. There were significant results in mean age difference between the beta-antagonist and the PG group [3.05 years, 95% confidence interval (CI) 1.54-4.57] and between the beta-antagonist group with the patients receiving a combined therapy (3.02 years, 95% CI 1.14-4.91). No significant difference was found between the PG and the combination group. All the three treated groups had a significant lower mean age than the control group at the time of cataract surgery.

**Conclusion:**

Based on our study, we concluded that there might be a possible association between chronic treatment with beta-antagonist agents and earlier cataract surgical time in the treated eye.

**Clinical significance:**

Intraocular pressure control is often usually achieved using ophthalmic agents. Their topical and systemic effects should be monitored precisely. Earlier cataract formation might be an important side effect which the physician has to keep in mind before choosing the suitable medication.

**How to cite this article:** Bontzos G, Agiorgiotakis M, Detorakis ET. Long-term Follow-up of Patients receiving Intraocular Pressure-lowering Medications as Cataract Surgery Candidates: A Case-control Study. J Curr Glaucoma Pract 2017;11(3):107-112.

## INTRODUCTION

Cataract is the opacification of the natural crystalline lens and breakdown of the lens protein microarchitecture, which adversely affects the transmission of light onto the retina and degrades optical quality.^1,2^ Cataract still remains a leading cause of visual impairment and blindness worldwide.^3-7^ The importance of risk factor identification for cataract development is evident and identifying strategies to prevent or delay cataractogenesis will be an essential part of clinical ophthalmic practice in the near future. Moreover, cataract may be concomitant with other ophthalmic morbidities, also affecting the aging human population, such as glaucoma.

Currently, intraocular pressure (IOP) reduction is the main goal of glaucoma treatment. Initial therapy is typically pharmaceutical, with topical ophthalmic agents^8-10^ such as PG analogues (e.g., tafluprost), p-adrenergic receptor antagonists (β-blockers e.g., timolol), a-adrenergic receptor agonists (a-agonists; e.g., brimonidine), carbonic anhydrase inhibitors (e.g., brinzolamide), and cholinergic receptor agonists (e.g., pilocarpine).^11^

The pharmacological mechanism of action of these agents varies. Beta-receptors are expressed throughout the eye, and their antagonists reduce aqueous humor production in the ciliary body by inhibiting synthesis of intracel-lular cyclic adenosine monophosphate.^11^ Topical carbonic anhydrase inhibitors reduce aqueous humor production by intervening in the carbonic anhydrase-dependent aqueous formation process.^11^ Prostaglandins accomplish ocular hypotensive effect by enhancing the uveoscleral outflow when binding to prostaglandin F (FP) receptors. The PGs have a minimal effect on aqueous humor production and episcleral venous pressure. They also modulate outflow facility through the trabecular outflow pathway. There are many supporting studies investigating PG mechanisms in the eye.^12-15^ Finally, a-agonists have a complex action in the aqueous turnover by intervening in both production and outflow mechanisms.^16,17^

Therefore, IOP-lowering medications alter the physiological aqueous humor secretion and outflow. Since the lens lacks blood vasculature, it receives all its nourishment from the aqueous humor. Nutrients diffuse in and out through the constant aqueous humor flow. Therefore, it should be reasonable to hypothesize that a disruption in lens homeostasis can eventually lead to cataract development. This study examines the potential association between long term antiglaucomatous drug therapy and cataract formation, with a view to estimate the added risk for cataract development in topical glaucoma medication users.

## MATERIALS AND METHODS

### Design

This study was designed as a retrospective assessment of patient data from hospital archives. All charts of patients who underwent first cataract surgery between January 1998 and December 2016 at the Department of Ophthalmology of the University Hospital of Heraklion, Crete, Greece, were reviewed and data from the first operated eye for cataract were obtained. Patients gave informed written consent for cataract surgery. All investigations analyzed in this study have been carried out in compliance with the Helsinki Declaration and were approved by our local ethics committee.

### Subjects

Patients who had been receiving topical IOP-lowering medication in the operated eye at the time of surgery were included for analysis. Patients were separated into three subgroups according to their medication treatment mechanism:

*Group I:* Under monotherapy with a β-blocker (timolol 0.5%),

*Group II:* Under monotherapy with a PG (latanoprost 0.005% or bimatoprost 0.03%)

*Group III:* Patients receiving combination of these medications (β-blocker + PG).

Inclusion criteria also were as follows: Patient age ≥60 years, primary open-angle glaucoma, and receiving the same medication (β-blocker, PG, or a combination treatment of these two) for at least 5 years before surgery. They were eligible for analysis if they presented with IOP values of <20 mm Hg in all their monitoring examinations before cataract extraction. Patients’ data were excluded from analysis if they had received other IOP-lowering medications like a-agonists or cholinergic receptor agonists at any time, reported with angle-closure glaucoma, congenital and traumatic cataracts, prior history of intraocular surgery,^18^ any history of inflammatory ocular disease,^19^ ocular infection or severe dry eye, and diabetes mellitus diagnosed for over a year before cataract surgery. These conditions can hasten the development of cataract as reported in multiple studies.^20-25^ Also, patients receiving topical or systemic corticosteroids for more than 30 days for any medical condition were also excluded.^26-28^ Finally, eyes with AL more than 28 mm were not included since AL greater than 30.0 mm has been associated with reduction in cataract age at surgery.^29^ Data from all non-glaucomatous patients who had cataract surgery and were age 60+ years at the time of their earliest cataract surgical procedure at the same department and during the same time interval were collected. The same exclusion criteria were followed for that control group.

The age at surgery, AL, as well as preoperative and postoperative (at the 6-month interval) BCVAs were recorded. Morphological information of the type and density of cataract (i.e., nuclear, cortical, subcapsular) was also included. In all, 320 patients were included providing a high observed statistical power throughout analysis; observed *post hoc* power was calculated by using Statistical Package for the Social Sciences (SPSS) version 22 and it was equal to 1.

### Statistical Analysis

The SPSS version 22.0 statistical package was used to generate graphs and to perform comparison tests between groups. All tests were two-tailed, and a p-value of 0.05 was determined to represent statistical significance. Normality for each of the four groups was verified by using Shapiro-Wilk test (group I: p = 0.53, group II: p = 0.183, and group III: p = 0.155, and general population group IV: p = 0.749, each one greater than a = 0.05). Applying Levene’s test for homogeneity of variances between groups showed that the variances are unequal (p = 0.0001). As the variances and the sample sizes differ, comparisons among the four groups were done using Welch’s robust test and Games-Howell *post hoc* test.

## RESULTS

**Graph 1:**
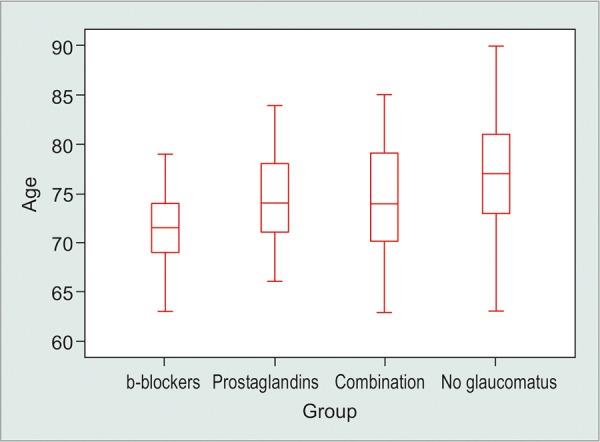
Box plots of age of cataract surgery for different subgroups which are separated based on different medications. Middle line in box represents the median age, lower box bound the first quartile, upper box bound the third quartile, whiskers the 95% confidence interval of the mean

The profile of this study is presented in Graph 1 and Table 1. In total, 320 patients were enrolled; 66 were receiving β-blocker topical medication, 98 were receiving PGs, and 78 were receiving a combined treatment of a β-blocker and a PG, either as two different drugs or as a fixed combination. Furthermore, 78 were nonglaucomatous patients who underwent cataract surgery and were assessed as a control group. Comparisons between different groups, standard deviations, and p-values are mentioned in Table 1.

The mean age when patients underwent first cataract surgery was 74.42 (SD = 5.055), and for each subgroup, the mean age is shown in Table 1.

There were statistical significant differences, concerning the age of cataract surgery between groups (β-blockers, PGs, combination, and nonglaucomatous groups; Welch’s robust test, p ≈ 0). For additional analysis between groups, Games-Howell *post hoc* test was applied. As seen in Table 2, there are significant differences for several comparisons and the p-value for each comparison is reported in Table 2. Comparing the three groups treated for glaucoma with the control group IV shows that mean differences are statistically significant with a younger mean age in the glaucomatous groups. The mean difference between the control and the β-blocker group is 5.88 (95% CI 3.90-7.86), while the control group and the PG group had a mean difference of 2.82 (95% CI 0.85-4.80).

Mean difference between control group and combination group was 2.85 (95% CI 0.59-5.12). Moreover, the mean age difference between β-blockers and PG groups was 3.05 (95% CI 1.54-4.57), and between β-blocker and combination group was 3.02 (95% CI 1.14-4.91), which implies that cataract progression is more rapid in patients treated with β-blockers.

Comparing the mean age of groups treated with PGs and combination treatment revealed no significant difference between the two group means (mean difference 0.03 with 95% CI -1.91-1.84).

## DISCUSSION

To the best of our knowledge, the association between treatment with antiglaucomatous β-blockers or PGs and the timing of cataract surgery has not been previously examined. Findings from this study imply that long-term antiglaucomatous treatment with specific β-blockers (timolol 0.5%) and PGs (latanoprost 0.005% or bimatoprost 0.03%) may lead to earlier cataract formation, compared with controls. A point of interest is that patients with glaucoma are in constant follow-up examinations. Cataract progression is monitored as a part of their ophthalmic examination allowing earlier detection and probably decision for extraction of a vision impairing cataract.

**Table 1:** **Table 1:** Age variation of cataract surgery for subgroups, based on treatment medication

								*95% CI for mean*							
*Variable*		*n*		*Mean age*		*Standard Deviation*		*Lower bound*		*Upper bound*		*Minimum*		*Maximum*	
Beta-blocker		66		71.3182		3.35655		70.4930		72.1433		63		79	
PG		98		74.3776		4.08799		73.5580		75.1971		66		84	
Combination		78		74.3462		5.24423		73.1638		75.5285		63		85	
Nonglaucomatous		78		77.2051		5.63007		75.9357		78.4745		63		90	
Total		320		74.4281		5.05529		73.8721		74.9841		63		90	

**Table 2:** **Table 2:** Comparison between mean age of each subgroup using Games-Howell *post hoc* test

								*95% CI*			
*(I) Medication*		*(J) Medication*		*Mean diff. (I-J)*		*p-value*		*Lower bound*		*Upper bound*	
Beta-blocker		PG		–3.05937*		0.000003		–4.5764		–1.5423	
		Combination		–3.02797*		0.000297		–4.9101		–1.1458	
		Nonglaucomatous		–5.88695*		0.000001		–7.8643		–3.9096	
PG		Beta-blocker		3.05937*		0.000003		1.5423		4.5764	
		Combination		0.03140		0.999970		–1.8487		1.9115	
		Nonglaucomatous		–2.82758*		0.001621		–4.8032		–0.8520	
Combination		Beta-blocker		3.02797*		0.000297		1.1458		4.9101	
		PG		–0.03140		0.999970		–1.9115		1.8487	
		Nonglaucomatous		–2.85897*		0.006933		–5.1218		–05961	
Nonglaucomatous		Beta-blocker		5.88695*		0.000001		3.9096		7.8643	
		PG		2.82758		0.001621		0.8520		4.8032	
		Combination		2.85897*		0.006933		0.5961		5.1218	

*The mean difference is significant at the 0.05 level

Glaucoma eye drop therapy would ideally maximize IOP-lowering efficacy and minimize adverse reactions. Several long-term topical and systemic side effects have been reported associated with IOP-lowering topical medications. For example, there are concerns regarding systemic side effects after beta-adrenoreceptor blocking activity in the pulmonary and circulatory system. Topical β-blocker may lower heart rate and blood pressure and may induce asthma and worsen chronic obstructive pulmonary diseases.^30-32^ Timolol drops have also been shown to decrease high-density lipoprotein and increase cholesterol. Diabetics may experience reduced glucose tolerance and hypoglycemic signs and symptoms can be masked.^33^ In addition, timolol induces a local anesthetic effect on the ocular surface, leading to poor tear secre-tion.^34^ Over time, chronic corneal toxicity from topical ocular medications may cause nerve damage, potentially resulting in neurotrophic keratopathy.

On the contrary, patients treated with topical PG analogues have a higher incidence of dry eye syndrome and Meibomian gland dysfunction.^35,36^ The PGs, and mostly latanoprost, have been associated with developing cystoid macular edema after been administered for ocular hypertension.^37^ Other benign side effects associated with PGs include eye pruritus, conjunctival hyperemia, periorbital lipodystrophy, and darkening of the iris, eyelashes, and periocular skin.^38^ In previous studies, PGs used as topical IOP-lowering agents have been questioned for their possible effects in crystalline lens homeostasis.^39^ Because lens epithelial cells express a high density of *FP* receptors, the mitogenic activities of PGs may alter lens physiology in long-term treatment.^39^ In short-term clinical use, the precise role of these PG receptors in lens epithelial cell pathophysiology has not been determined.

Strengths of our study include its long-term follow-up of patient medical records with reasonable rates of surveillance, consistency of the statistical methods used, and masked judging of the age at cataract surgery. Furthermore, patients and controls were recruited from the same population, which increases the consistency of the results. Glaucomatous patients were in close follow-up and their frequent slit-lamp examination enabled early detection of the cataract progression. However, there are also limitations to be mentioned. This study as a retrospective analysis cannot demonstrate causation. The analysis was conducted as a single-center study with potential subjective bias in surgical decisions. Moreover, since the patients were already in senile age group, the fact that they already had lens changes cannot be denied.

Ideally, young patients receiving IOP-lowering treatment should be recruited. Thus, cataract formation can be estimated irrespective of normal lens aging.

The effects of drug instillation frequency and preservatives are not examined in this study. The cataract morphological characteristics were not further correlated with the timing of cataract extraction.

Moreover, this study did not specifically look at other concomitant conditions which may predispose to glaucoma development and also affect the lens by histochemi-cal changes or hemodynamic alterations at the anterior segment, such as pseudoexfoliation syndrome.^40^ The pathomechanism underlying any potential association between antiglaucomatous therapy and cataract formation remains unclear. However, some possible explanations might be speculated; a reduction in aqueous humor production can result in reduced oxygen supplies for lens metabolic needs. Since cataract etiology is not fully understood, shifts in aqueous humor hydrodynamics and its association with cataract development may lead to more insights into the underlying mechanisms of cataract disease.

## CONCLUSION

In summary, findings from our analysis add indirect evidence to the hypothesis that chronic topical β-blocker use may increase the risk of cataract formation. In future research, prospective, randomized trials are needed to examine the effect of IOP-lowering medication and cataract formation and progression. Since the pharmacological toxicity of antiglaucomatous medications may be cumulative, it is important to examine the time interval during which the patients are exposed and its potential correlation with cataract development.

## CLINICAL SIGNIFICANCE

Our study findings suggest that the risk of developing a cataract should be taken under consideration when accessing a patient on topical antiglaucoma drug. Patients should be carefully evaluated regarding their age and overall health when first administered with an antiglau-comatous agent.

## References

[1] Asbell PA, Dualan I, Mindel J, Brocks D, Ahmad M, Epstein S (2005). Age-related cataract. Lancet..

[2] Leibowitz HM, Krueger DE, Maunder LR, Milton RC, Kini MM, Kahn HA, Nickerson RJ, Pool J, Colton TL, Ganley JP (1980). The Framingham Eye Study monograph: an ophthalmo-logical and epidemiological study of cataract, glaucoma, diabetic retinopathy, macular degeneration, and visual acuity in a general population of 2631 adults, 1973-1975. Surv Ophthalmol.

[3] Hyman L, Wu SY, Connell AM, Schachat A, Nemesure B, Hennis A, Leske MC (2001). Prevalence and causes of visual impairment in The Barbados Eye Study. Ophthalmology.

[4] Varma R, Ying-Lai M, Klein R, Azen SP (2004). Prevalence and risk indicators of visual impairment and blindness in Latinos: the Los Angeles Latino Eye Study. Ophthalmology.

[5] Zhao J, Ellwein LB, Cui H, Ge J, Guan H, Lv J, Ma X, Yin J, Yin ZQ, Yuan Y (2010). Prevalence of vision impairment in older adults in rural China: the China Nine-Province Survey. Ophthalmology.

[6] Murthy GV, Gupta S, Ellwein LB, Munoz SR, Bachani D, Dada VK (2001). A population-based eye survey of older adults in a rural district of Rajasthan: I. Central vision impairment, blindness, and cataract surgery. Ophthalmology.

[7] Zheng Y, Lavanya R, Wu R, Wong WL, Wang JJ, Mitchell P, Cheung N, Cajucom-Uy H, Lamoureux E, Aung T (2011). Prevalence and causes of visual impairment and blindness in an urban Indian population: the Singapore Indian eye study. Ophthalmology.

[8] European Glaucoma Society. Terminology and guidelines for glaucoma. 4th ed. 2014 Available from:. http://www.eugs.org/.

[9] Aptel F, Chiquet C, Romanet J-P (2012). Intraocular pressure-lowering combination therapies with prostaglandin analogues. Drugs.

[10] Irkec M, Bozkurt B, Mocan MC (2013). Are preservatives necessary to improve efficacy of some glaucoma drops?. Br J Ophthalmol.

[11] Schmidl D, Schmetterer L, Garhofer G, Popa-Cherecheanu A (2015). Pharmacotherapy of glaucoma. J Ocul Pharmacol Ther.

[12] Brubaker RF, Schoff EO, Nau CB, Carpenter SP, Chen K, Vandenburgh AM (2001). Effects of AGN 192024, a new ocular hypotensive agent, on aqueous dynamics. Am J Ophthalmol.

[13] Weinreb RN, Toris CB, Gabelt BT, Lindsey JD, Kaufman PL (2002). Effects of prostaglandins on the aqueous humor outflow pathways. Surv Ophthalmol.

[14] Christiansen GA, Nau CB, McLaren JW, Johnson DH (2004). Mechanism of ocular hypotensive action of bimatoprost (Lumigan) in patients with ocular hypertension or glaucoma. Ophthalmology.

[15] Lim KS, Nau CB, O’Byrne MM, Hodge DO, Toris CB, McLaren JW, Johnson DH (2008). Mechanism of action of bima-toprost, latanoprost, and travoprost in healthy subjects. A crossover study. Ophthalmology.

[16] Potter DE, Crosson CE, Heath AR, Ogidigben MJ (1990). Review: alpha 2 and DA2 agonists as antiglaucoma agents: comparative pharmacology and clinical potential. J Ocul Pharmacol.

[17] Greenfield DS, Liebmann JM, Ritch R (1997). Brimonidine: a new alpha2-adrenoreceptor agonist for glaucoma treatment. J Glaucoma.

[18] Feng H, Adelman RA (2014). Cataract formation following vitreo-retinal procedures. Clin Ophthalmol.

[19] Foster CS, Barrett F (1993). Cataract development and cataract surgery in patients with juvenile rheumatoid arthritis-associated iridocyclitis. Ophthalmology.

[20] Delcourt C, Cristol JP, Tessier F, Leger CL, Michel F, Papoz L (2000). Risk factors for cortical, nuclear, and posterior subcapsular cataracts: the POLA study. Pathologies Oculaires Lieesal’Age. Am J Epidemiol.

[21] Leske MC, Chylack LT Jr, Wu SY (1991). The lens opacities case-control study. Risk factors for cataract. Arch Ophthalmol.

[22] Mukesh BN, Le A, Dimitrov PN, Ahmed S, Taylor HR, McCarty CA (2006). Development of cataract and associated risk factors: the Visual Impairment Project. Arch Ophthalmol.

[23] Hennis A, Wu SY, Nemesure B, Leske MC (2004). Risk factors for incident cortical and posterior subcapsular lens opacities in the Barbados Eye Studies. Arch Ophthalmol.

[24] Klein BE, Klein R, Lee KE (1998). Diabetes, cardiovascular disease, selected cardiovascular disease risk factors, and the 5-year incidence of age-related cataract and progression of lens opacities: the Beaver Dam Eye Study. Am J Ophthalmol.

[25] Tan JS, Wang JJ, Mitchell P (2008). Influence of diabetes and cardiovascular disease on the long-term incidence of cataract: the Blue Mountains Eye Study. Ophthalmic Epidemiol.

[26] Cumming RG, Mitchell P, Leeder SR (1997). Use of inhaled corti-costeroids and the risk of cataracts. N Engl J Med.

[27] Wang JJ, Rochtchina E, Tan AG, Cumming RG, Leeder SR, Mitchell P (2009). Use of inhaled and oral corticosteroids and the long-term risk of cataract. Ophthalmology.

[28] Urban RC, Cotlier R (1986). Corticosteroid-induced cataracts. Surv Ophthalmol.

[29] Tuft SJ, Bunce C (2004). Axial length and age at cataract surgery. J Cataract Refract Surg.

[30] Stewart WC, Stewart JA, Jackson AL (2002). Cardiovascular effects of timolol maleate, brimonidine or brimonidine/timolol maleate in concomitant therapy. Acta Ophthalmol Scand.

[31] Schuman JS (2000). Effects of systemic beta-blocker therapy on the efficacy and safety of topical brimonidine and timolol. Brimonidine Study Groups 1 and 2. Ophthalmology.

[32] Maclure GM (1983). Chronic open angle glaucoma treated with Timolol. A four year study. Trans Ophthalmol Soc UK.

[33] Mitchell P, Wang JJ, Cumming RG, House P, England JD (2000). Long-term topical timolol and blood lipids: the Blue Mountains Eye Study. J Glaucoma.

[34] Weissman SS, Asbell PA (1990). Effects of topical timolol (0.5%) and betaxolol (0.5%) on corneal sensitivity. Br J Ophthalmol.

[35] Mehta M, Siddique SS, Gonzalez-Gonzalez LA, Foster CS (2011). Immunohistochemical differences between normal and chronically inflamed conjunctiva: diagnostic features. Am J Dermatopathol.

[36] Henriksson JT, De Paiva CS, Farley W, Pflugfelder SC, Burns AR, Bergmanson JP (2013). Morphologic alterations of the palpebral conjunctival epithelium in a dry eye model. Cornea.

[37] Ayyala RS, Cruz DA, Margo CE, Harman LE, Pautler SE, Misch DM, Mines JA, Richards DW (1998). Cystoid macular edema associated with latanoprost in aphakic and pseudophakic eyes. Am J Ophthalmol.

[38] Filippopoulos T, Paula JS, Torun N, Hatton MP, Pasquale LR, Grosskreutz CL (2008). Periorbital changes associated with topical bimatoprost. Ophthal Plast Reconstr Surg.

[39] Mukhopadhyay P, Bhattacherjee P, Andom T, Geoghegan TE, Andley UP, Paterson CA (1999). Expression of prostaglandin receptors EP4 and FP in human lens epithelial cells. Invest Ophthalmol Vis Sci.

[40] Detorakis ET, Achtaropoulos AK, Drakonaki EE, Kozobolis VP (2007). Hemodynamic evaluation of the posterior ciliary circulation in exfoliation syndrome and exfoliation glaucoma. Graefes Arch Clin Exp Ophthalmol.

